# Rolling predictive optimal scheduling of reservoirs for flood control and power generation under prediction uncertainty

**DOI:** 10.1038/s41598-026-43532-6

**Published:** 2026-03-24

**Authors:** Zhongzheng He, Jun Guo, Zhiming Cao, Yongqiang Wang, Chao Wang, Hui Qin

**Affiliations:** 1 School of Civil Engineering and Architecture, Nanchang University, Nanchang, 330031 China; 2 Key Laboratory of Poyang Lake Environment and Resource Utilization, Ministry of Education, Nanchang University, Nanchang, 330031 China; 3 Changjiang River Scientific Research Institute, Wuhan, 430015 China; 4 China Institute of Water Resources and Hydropower Research, Beijing, 100038 China; 5 School of Civil and Hydraulic Engineering, Huazhong University of Science and Technology, Wuhan, 430074 China; 6 Hubei Key Laboratory of Digital Valley Science and Technology, Huazhong University of Science and Technology, Wuhan, 430074 China

**Keywords:** Rolling forecast optimization scheduling, Flood control scheduling, Power generation scheduling, Flood prediction period, Flood control water level, Hydrology, Natural hazards

## Abstract

The Xiajiang Reservoir, located in the middle Ganjiang River of the Yangtze River Basin, requires scientific inflow prediction and optimised scheduling to balance the flood control and power generation. Traditional methods are constrained by an overreliance on historical data, insufficient flood samples, and poor high-flow performance, necessitating improved models. Focusing on the Xiajiang Reservoir, this study developed a multi-model series–parallel coupling inflow prediction model to test different coupling modes and a rolling prediction optimisation scheduling model integrated with the inflow model. Using typical design floods as input, we analysed the impacts of the flood forecast period and dynamic flood control water level on benefits and explored performance across flow scenarios. The key results showed that the parallel coupling framework achieved excellent short-term prediction, whereas the series coupling model had obvious limitations, and the series–parallel model underperformed. The rolling scheduling modelwith dynamically updated hydrological dataeffectively balances the two objectives. Within 72 h, the maximum outflow first decreased and then stabilised; moderately raising the dynamic flood control water level boosted power generation with minimal flood risk. Additionally, traditional forecasting struggled with high flows because of scarce samples, whereas abundant low-flow samples ensured higher accuracy and better scheduling results. This study strengthens the scientific basis for inflow prediction and scheduling in the Xiajiang Reservoir and provides a valuable reference for similar reservoirs in the Yangtze River Basin.

**Supplementary Information:**

The online version contains supplementary material available at 10.1038/s41598-026-43532-6.

## Introduction

Water resources are crucial for human survival; their imbalance leads to floods or droughts^1^. Reservoirs play a vital role in regulating water distribution and addressing uneven spatiotemporal water resource issues^2,3^. They also serve multiple functions, such as flood control, navigation, power generation, and irrigation ^4–6^. Therefore, optimising reservoir scheduling, balancing flood control and power generation, and balancing flood control and water resource utilisation during the flood season are crucial.

Scholars worldwide have conducted extensive research on reservoir flood control scheduling optimisation, proposing optimal seasonal storage models, water storage expansion methods, simulation-based DC-FLWL models for cascade reservoirs, two-stage risk analysis, opportunity-constrained optimal control, and multi-objective risk management models. However, these lack a systematic integration of multi-objective strategies, prediction-scheduling coupling, and flood scenario adaptability. Advances in GIS, rainfall radar, and satellite cloud map technologies have enhanced flood forecasting accuracy, driving real-time forecasting optimisation scheduling research, with relevant studies including Mixed Integer Linear Programming (MILP)-Bayesian Network (BN) integrated models, collaborative optimisation combining particle swarm optimisation (PSO), progressive optimisation algorithm (POA), and Dynamic Programming (DP), and dispatch systems emphasising flood control level-forecasting technology links.Some studies have pointed out that the multi-objective coupling and complex conditions in reservoir operation models will dynamically change with the operating cycle and environmental changes, and reservoir operation plans need to adapt to the latest environmental data^7^.

At the same time, many achievements have emerged in the research of reservoir flood control optimization and conventional scheduling under uncertain inflow scenarios. Such as studying the uncertainty of inflow forecasting^8,9^, uncertainty in water demand forecasting^10,11^, and the dual uncertainty of inflow demand^12–15^; A risk analysis model for reservoir flood management and scheduling based on Bayesian networks has been constructed^16^; the framework of "parameter simulation optimization" has been explored; reservoir prediction and scheduling rules have been developed by combining net rainfall processes and uncertain inflow processes; a reservoir flood control risk analysis method that considers multiple uncertainties has been developed^17^; and a multi-time scale risk analysis method for hydropower reservoirs has been proposed ^18^. However, various reservoir scheduling methods that consider uncertainty have their own advantages and disadvantages^19^. Although explicit stochastic optimisation scheduling can comprehensively reflect the impact of uncertainty and obtain the optimal strategy in a probabilistic sense, it relies heavily on data and is computationally complex^20^. Implicit stochastic optimisation scheduling is relatively simple and has lower requirements for data and computation; however, it is not precise and comprehensive enough when dealing with uncertainty, especially in multi-reservoir systems where a large number of inflow scenarios and deterministic optimisation problems need to be solved, and appropriate post-processing methods are required to infer operating rules^21,22^. Predictive optimisation scheduling can flexibly adjust plans based on real-time prediction information; however, it is constrained by the accuracy of inflow prediction models and the accumulation of prediction errors over time, which affect the stability and reliability of scheduling decisions^23,24^. In addition, these studies mainly focus on optimising scheduling methods or integrating technologies, with insufficient comparative analysis of the adaptability of different optimisation algorithms and coupling effects of forecasting-scheduling models under dynamic environmental changes, leaving the exploration of more robust real-time scheduling frameworks to be further advanced.

Despite the above progress, the mechanisms of factors such as foresight period, variable control, and scheduling objectives in the rolling forecasting optimization scheduling mode of reservoirs are still unclear, and there is a lack of systematic analysis of the many factors that affect the flood control and power generation benefits of reservoirs. This limits the application of rolling forecasting optimization scheduling technology in practical engineering.

Based on this, this study takes Xiajiang Reservoir as the object, with core innovations and content as follows:

(1) To address the poor adaptability of existing single-coupling-mode prediction models, a multi-model series–parallel coupling correction inflow prediction model was constructed (integrating BP, SVM, LSTM, GPR, MLR, RT, and three coupling modes). Systematic comparison verifies the parallel framework outperforms serial/series–parallel modes in short-term prediction with higher accuracy and stability.(2) Addressing insufficient dynamic coupling between prediction and scheduling in existing research, a dual-objective (flood control+power generation) reservoir rolling prediction optimization scheduling model was established—adopting dynamically updated hydrological data, rolling time window coupling, and POA algorithm.(3) Aiming at unclear regulatory mechanisms of key factors in rolling scheduling, 16 typical design floods were used to quantitatively analyze flood forecast period and dynamic flood control water level impacts.(4) Solving scenario adaptability flaws of traditional historical data-dependent forecasting, this study reveals prediction-scheduling performance differences between high/low-flow scenarios: scarce high-flow samples cause poor accuracy, while abundant low-flow samples ensure reliable scheduling, providing targeted optimization directions.

The remainder of this paper is structured as follows: Sect. 2 introduces data-driven short-term runoff prediction methods. Section 3 constructs a reservoir-rolling prediction optimisation-scheduling model. Section 4 uses the Xiajiang Reservoir as a case study to analyse the coupling correction effects, influencing factors, and performance under high/Low inflow scenarios. Section 5 discusses the results, proposes optimisation strategies, and outlines the limitations and future directions. Section 6 summarises the findings, emphasising the significance of the model framework and the importance of benefit balance and strategy optimisation.

## Methodology

### Short-term runoff forecasting based on data-driven methods

#### Single-model runoff prediction method

**Fig. 1:**
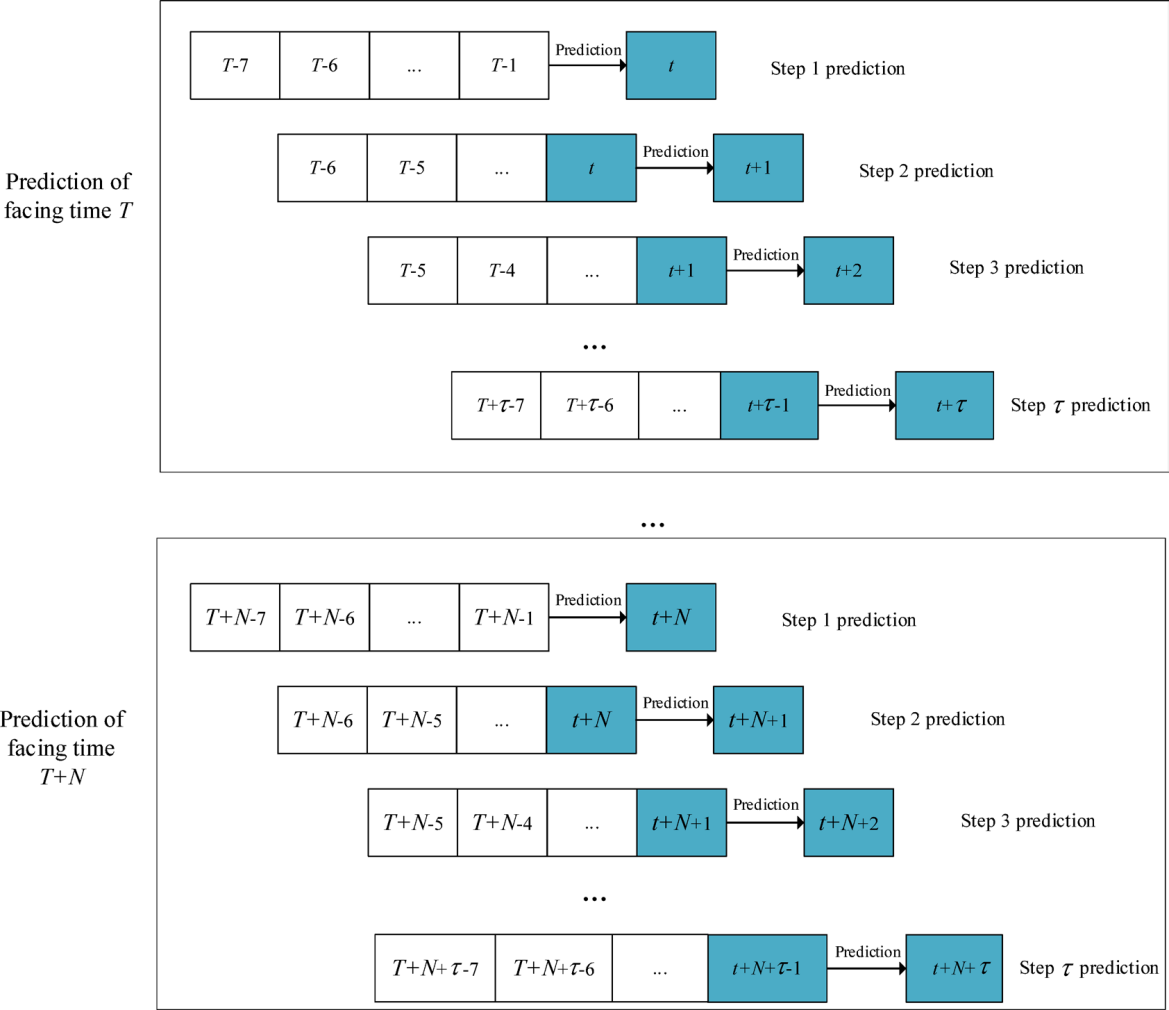
Multi step rolling prediction process of runoff based on data driven.

This study selects six typical data-driven models as representatives for runoff prediction research, including BP, SVM, LSTM, GPR, MLR, and RT. These six models are used to establish single-step runoff forecasting models.. Subsequently, a multi-step prediction framework was constructed based on a rolling forecasting approach, which relies on a sliding time window to achieve full coverage of N total periods via progressive prediction. With a 6-h time step, seven historical data points were selected as forecast factors to predict the runoff in the next period. The rolling multi-step runoff prediction process is shown in Fig. 1.

#### Parallel correction model for runoff prediction

As hydrological prediction research progresses continuously, a plethora of prediction models keep emerging. It is crucial to recognize that distinct models have well—defined application scopes, and there are substantial discrepancies in their prediction accuracies when applied to the same hydrological time series^25^.

**Fig. 2:**
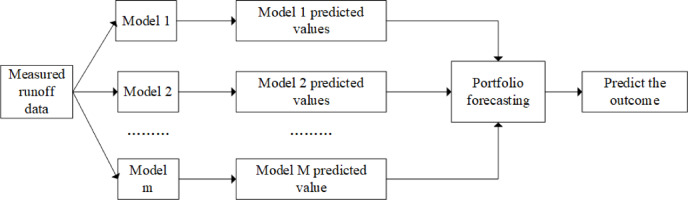
Flow chart of parallel correction method.

To enhance prediction precision, this section employs a multi—model fusion strategy for the collaborative prediction of the target hydrological sequence^26^. Through the establishment of a weight—allocation mechanism, it effectively harnesses the strengths of each model while mitigating the influence of their inherent limitations on the outcomes. This integrated approach not only minimizes the overall prediction error and alleviates the contention regarding the selection of a single optimal model, but also concurrently elevates both the accuracy and stability of runoff prediction results. The specific process of the parallel correction method is shown in Fig. 2.

Based on the differences in the calculation methods of the weight coefficients, the parallel combination correction model for runoff prediction can be divided into two types: optimal and non-optimal. The core solution logic of the optimal parallel model is to determine the optimal weight coefficients corresponding to each individual prediction model involved in the coupling by solving the extremum of the objective function under specified constraint conditions. Based on this solution logic, the mathematical expression of the optimal parallel model can be described as:

1 $$\begin{gathered} \max (\min )Q = Q(w_{1} ,w_{2} , \ldots ,w_{m} )\quad \hfill \\ s.t.\quad \left\{ {\begin{array}{*{20}l} {\sum\limits_{i = 1}^{m} {w_{i} } = 1} \hfill \\ {w_{i} \ge 0,i = 1,2, \ldots ,m} \hfill \\ \end{array} } \right. \hfill \\ \end{gathered}$$

Among them, $Q(w_{1} ,w_{2} , \ldots ,w_{m} )$ is the desired objective function; $(w_{1} ,w_{2} , \ldots ,w_{m} )$ is the optimal weight coefficients for each model; $m$ Indicate the type or number of prediction models.

The parallel combination correction model adopts common strategies, including the arithmetic mean, inverse sum of squared prediction errors, inverse mean square error, weighted mean, and binomial coefficient methods. Numerical calculations demonstrated a significant accuracy gap between these methods and the optimised combination prediction method; thus, an optimal parallel grouping method was developed to construct the prediction model.

### Series correction method for runoff prediction

**Fig. 3:**
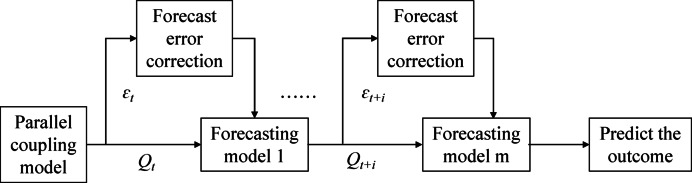
Flow chart of serial correction method.

Existing hydrological models fail to accurately depict complex natural hydrological processes. Thus, prediction errors remain inevitable, even with parallel predictions using the six models above. Real-time correction utilises existing hydrological conditions to revise future forecast values and reduce errors, with its core relying on “new information” for real-time model adjustments. However, owing to the uncertainty in the model input, model structure, and model parameters, hydrological models may generate certain forecast errors when conducting hydrological forecasting^27^.Because the causes of forecast errors (systematic and random) cannot be distinguished, real-time correction methods lack a strict hydrological physical basis, and common methods rely on mathematics, statistics, and modern automatic control theories. Among them, the flowchart of serial correction is shown in the Fig. 3.Among them, $Q_{t + i}$ is runoff data, and $\varepsilon_{t + i}$ is forecast error.

Research shows that deterministic time series functions cannot accurately describe the uncertainty fluctuation characteristics of hydrological element time series, but probabilistic analytical methods can establish suitable dynamic statistical models to simulate evolution laws. Among the six selected models, GPR was chosen to predict runoff prediction errors and realise real-time dynamic compensation. Notably, the constructed predictive architecture relies entirely on endogenous time series observation data, breaking through the theoretical constraints of independent variable distribution in traditional models and effectively avoiding feature selection bias and covariate linear dependence in conventional regression analyses. The expression of GPR error prediction model is:

2 $$X_{t} = b_{1} X_{t - 1} + b_{2} X_{t - 2} + \cdots b_{n} X_{t - n} + \xi$$

Among them:

$Y = [x_{n + 1} {\kern 1pt} {\kern 1pt} x_{n + 2} {\kern 1pt} \cdots {\kern 1pt} {\kern 1pt} x_{N} ]^{T}$ is a series of observed values; $X_{t - 1} ,X_{t - 2} , \cdots ,X_{t - n}$ is the observation value of the same stationary sequence for several periods; $b_{1} ,b_{2} , \cdots ,b_{n}$ is the regression parameter; $\xi$ has a mean of 0; A white noise signal with a variance of a certain value.

Assuming an observation value of $X_{t} (t = 1,2, \cdots ,N)$, note:

3 $$Y = [x_{n + 1} {\kern 1pt} {\kern 1pt} x_{n + 2} {\kern 1pt} \cdots {\kern 1pt} {\kern 1pt} x_{N} ]^{T}$$

4 $$\xi = [\xi_{t - 1} {\kern 1pt} {\kern 1pt} \xi_{t - 1} \cdots \xi_{t - n} ]^{T}$$

5 $$B = [b_{1} {\kern 1pt} {\kern 1pt} {\kern 1pt} b_{2} {\kern 1pt} {\kern 1pt} \cdots b_{n} ]^{T}$$

6 $$X = \left[ \begin{gathered} x_{n} {\kern 1pt} {\kern 1pt} {\kern 1pt} {\kern 1pt} {\kern 1pt} {\kern 1pt} {\kern 1pt} {\kern 1pt} {\kern 1pt} x_{n - 1} \cdots {\kern 1pt} {\kern 1pt} {\kern 1pt} x_{1} \hfill \\ x_{n + 1} {\kern 1pt} {\kern 1pt} {\kern 1pt} {\kern 1pt} x_{n} {\kern 1pt} {\kern 1pt} {\kern 1pt} {\kern 1pt} {\kern 1pt} {\kern 1pt} \cdots {\kern 1pt} {\kern 1pt} {\kern 1pt} {\kern 1pt} x_{2} \hfill \\ \vdots {\kern 1pt} {\kern 1pt} {\kern 1pt} {\kern 1pt} {\kern 1pt} {\kern 1pt} {\kern 1pt} {\kern 1pt} {\kern 1pt} {\kern 1pt} {\kern 1pt} {\kern 1pt} {\kern 1pt} {\kern 1pt} {\kern 1pt} {\kern 1pt} {\kern 1pt} {\kern 1pt} {\kern 1pt} {\kern 1pt} {\kern 1pt} {\kern 1pt} \vdots {\kern 1pt} {\kern 1pt} {\kern 1pt} {\kern 1pt} {\kern 1pt} {\kern 1pt} {\kern 1pt} {\kern 1pt} {\kern 1pt} {\kern 1pt} {\kern 1pt} {\kern 1pt} {\kern 1pt} {\kern 1pt} {\kern 1pt} {\kern 1pt} \vdots {\kern 1pt} {\kern 1pt} {\kern 1pt} {\kern 1pt} {\kern 1pt} {\kern 1pt} {\kern 1pt} {\kern 1pt} {\kern 1pt} {\kern 1pt} {\kern 1pt} {\kern 1pt} \vdots \hfill \\ x_{N - 1} {\kern 1pt} x_{N - 2} {\kern 1pt} {\kern 1pt} {\kern 1pt} {\kern 1pt} {\kern 1pt} {\kern 1pt} {\kern 1pt} {\kern 1pt} {\kern 1pt} {\kern 1pt} x_{N - n} {\kern 1pt} \hfill \\ \end{gathered} \right]$$

The final error prediction model can be expressed as:

7 $$Y = XB + \xi$$

### Model calibration and validation

To ensure the reliability and generalisation ability of the constructed short-term runoff prediction models, standardised data division and model-training procedures were formulated.

### Dataset splitting

To avoid data leakage and conform to hydrological temporal continuity, the dataset was split by time sequence: the first 80% was the training set and the remaining 20% was the test set. All data were unified to a 6-h time step, matching the reservoir’s actual scheduling cycle and the designed rolling prediction window. Data preprocessing ensured quality: short-term missing values were supplemented by linear interpolation, and abnormal values were corrected using hydrological constraints.

### Model training

Single models: Key hyperparameters were optimised via fivefold cross-validation on the training set. Training stopped when the validation error stabilised or the maximum epochs were reached, with regularisation (e.g. dropout) or stepwise regression to avoid overfitting.

#### Coupling models

*Parallel correction model*: Optimised weights of pre-trained single models for minimum error using POA.

*Series correction model*: An error prediction model was trained with base model training errors to dynamically compensate for base predictions.

*Series–parallel coupling model*: Took the parallel model as the front-end, corrected its errors with the pre-trained error model, and trained the entire structure end-to-end on the training set.

### Evaluation index of prediction model

To achieve a more targeted quantitative evaluation of forecasting performance, this study selected four accuracy verification indicators: Mean Absolute Error (MAE), Root Mean Squared Error (RMSE), Deterministic Coefficient (DC), and Qualified Rate (QR) as the benchmarks for measuring forecasting effects.

MAE intuitively reflects prediction accuracy; smaller values indicate that the predictions are closer to the true values. The RMSE characterizes the overall deviation between the predicted and measured data; a lower value indicates a reduced comprehensive error. The DC ranges from [0, 1], with values closer to 1 signifying a stronger linear correlation between the predicted and actual values. The QR ranges from [0, 1], quantifying the prediction reliability; a higher value directly reflects improved predictive performance. The calculation formulas of each indicator are shown below:

8 $$RMSE = \sqrt {\frac{1}{N}\sum\limits_{i = 1}^{N} {(S_{i} - O_{i} )^{2} } }$$

9 $$MAE = \frac{1}{N}\Sigma_{i = 1}^{N} |S_{i} - O_{i} |$$

10 $$DC = 1 - \frac{{\sum\limits_{i = 1}^{N} {(S_{i} - O_{i} )^{2} } }}{{\sum\limits_{i = 1}^{N} {(O_{i} - \overline{O}_{i} )^{2} } }}$$

11 $$QR = \frac{k}{n} \times 100\%$$

where $S_{i}$ is the simulated value; $O_{i}$ is the measured value; $\overline{O}_{i \, }$ is the mean value of the measured values; $k$ is the number of samples with a relative error less than 20% in the sample; $n$ is the number of test samples.

## Reservoir rolling forecast optimization scheduling model

### A mathematical model for real-time rolling forecasting and optimization scheduling of reservoirs

In reservoir scheduling, multi-objective coordination is essential, requiring a multi-objective optimal scheduling model to develop short-term plans and real-time directives based on the projected inflow. During the flood season, the model balances flood control and power generation by maximising the power output while meeting peak flood reduction requirements and ensuring effective flood control. Unlike deterministic optimal scheduling relying on known inflow, the real-time rolling forecast optimal scheduling model only considers inflow forecasting within a restricted horizon, better reflecting actual reservoir operation and management. Assuming the effective forecast horizon for incoming water is $\tau$ time period, the real-time rolling forecast optimal scheduling model of the reservoir is adjusted from a comprehensive process optimization to an objective optimization within the effective forecast horizon ($t \to t + \tau$ time period), represented by the following equation.

12 $$Obj_{1} :\min \, Q_{t \to t + \tau }^{\max } = \min \left\{ {\max \left\{ {Q_{t}^{{}} ,Q_{t + 1}^{{}} ,Q_{t + 2}^{{}} ,...,Q_{t + \tau }^{{}} } \right\}} \right\}{, }t = t,t + 1,...,t + \tau$$

13 $$Obj_{2} :\max E_{t \to t + \tau }^{{}} = \sum\limits_{t = t}^{t + \tau } {N_{t} \Delta T_{t} } = \sum\limits_{t = 1}^{t + \tau } {KG_{t} H_{t} \Delta T_{t} }$$

where $Q_{t - 1}^{{}}$ and $Q_{t}^{{}}$ are the outflow discharge of the reservoir at the beginning of the $t$ period, m^3^/s; $Q_{t \to t + \tau }^{\max }$ represents the maximum discharge flow (m3/s) at the commencement of time period $t$ to $t + \tau$; $E_{t \to t + \tau }^{{}}$ denotes the total power generation (10,000 kWh) at the onset of time period $t$ to $t + \tau$; *K* signifies the integrated output coefficient of the reservoir power generation; $N_{t}$ indicates the output during time period $t$ (10,000 kWh); $G_{t}$ refers to the power generation flow (m3/s) during time period $t$; $H_{t}$ is the average head of netting in time period $t$ (m); and $\Delta T_{t}$ represents the duration of the time period in time period $t$.

Moreover, some constraints regarding dam safety must be considered in the best scheduling of reservoir flood control.

1. Water balance
  14 $$V_{t} = V_{t - 1} + (I_{t - 1} - \frac{{Q_{t - 1} + Q_{t} }}{2}) \cdot \Delta T$$
  15 $$\frac{{Q_{t - 1} + Q_{t} }}{2} = G_{t} + S_{t}$$

(2) Storage level constraints
  16 $$Z_{t}^{\min } \le Z_{t} \le Z_{t}^{\max }$$
  17 $$V_{t}^{\min } \le V_{t} \le V_{t}^{\max }$$

(3) Flow constraints
  18 $$Q_{t}^{\min } \le Q_{t} \le Q_{t}^{\max }$$

(4) Boundary constraints
  19 $$Z_{0} = Z_{begin} ,Z_{T} = Z_{end}$$
  where $I_{t}$ and $S_{t}$ are the incoming and outgoing water flow in time period $\tau$, m^3^/s; $Z_{t}^{\min }$ and $Z_{t}^{\max }$ are the water level constraints in time period $t$, m; $V_{t}^{\min }$ and $V_{t}^{\max }$ are the reservoir capacity constraints in time period $t$, billion m^3^; $Q_{t}^{\min }$ and $Q_{t}^{\max }$ are the outflow discharge constraints in time period $t$, m^3^/s; $Z_{i}^{begin}$ and $Z_{i}^{end}$ are the control water level at the beginning and end of the dispatch period, (m).

**Fig. 4:**
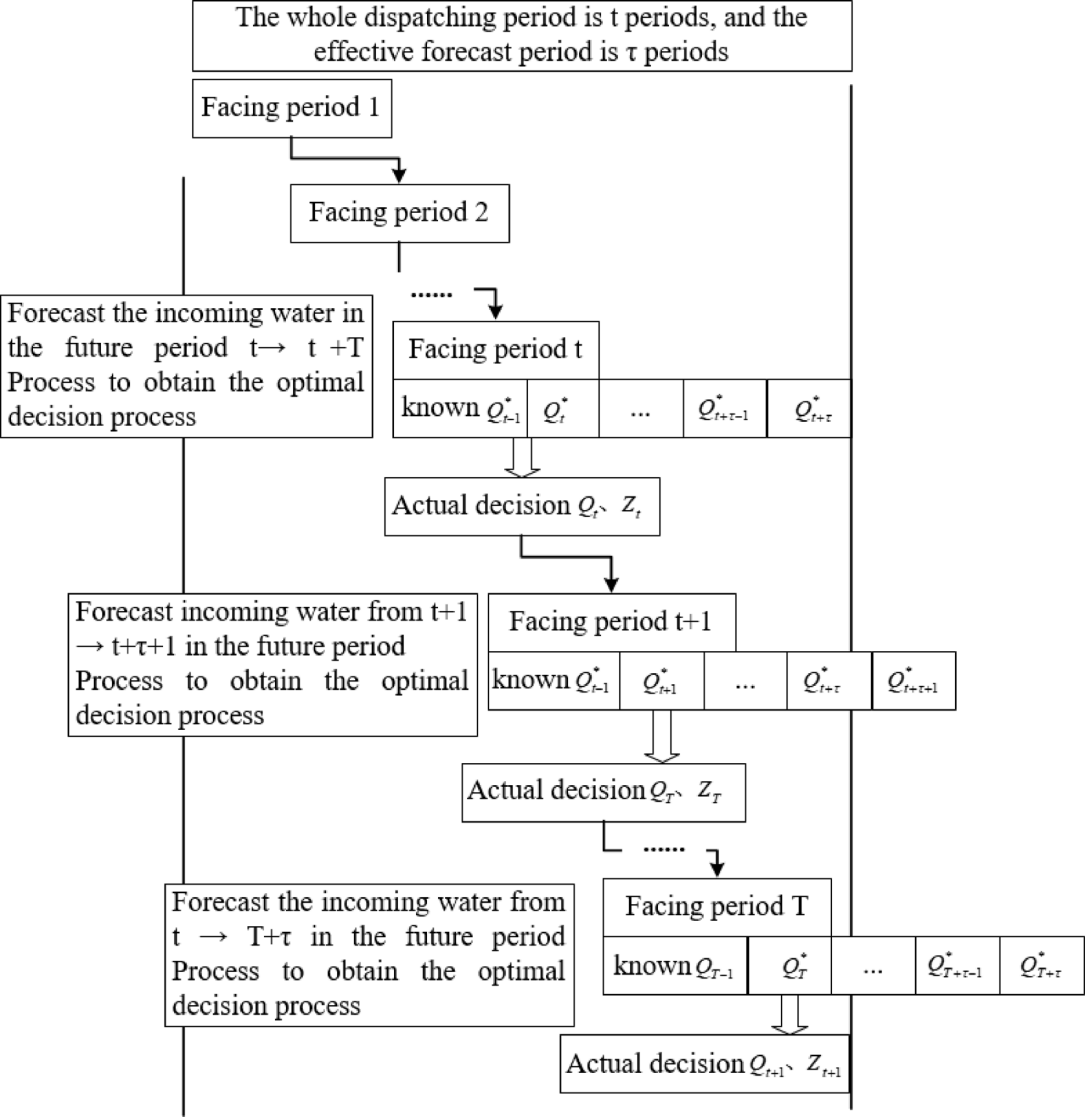
Schematic diagram of the real-time rolling forecast optimization scheduling of the reservoir.

**Fig. 5:**
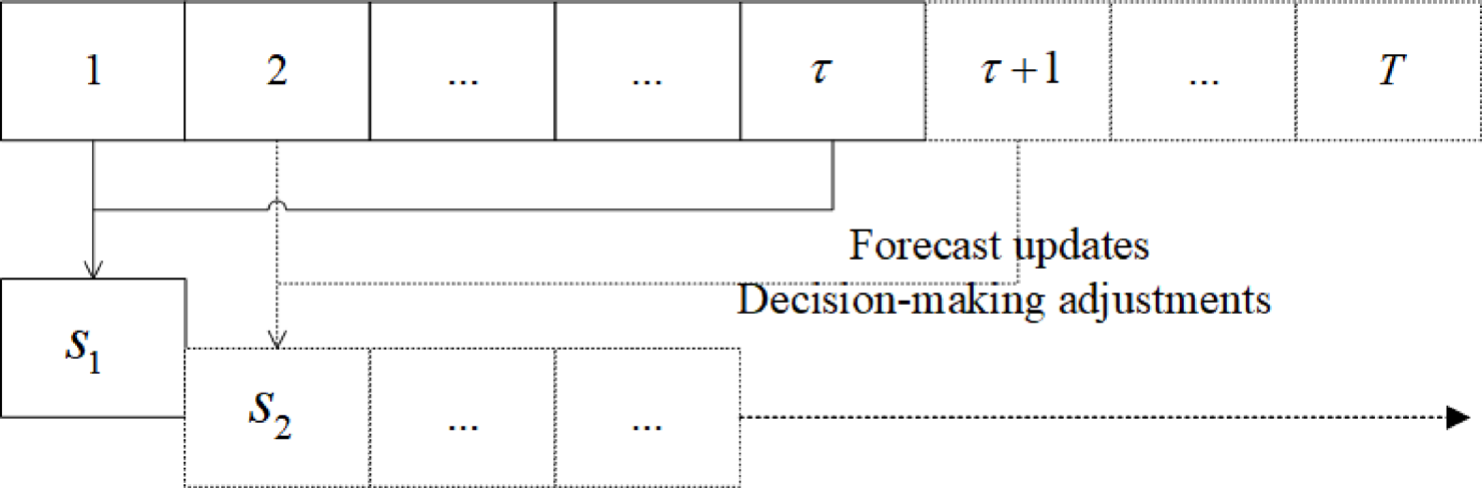
Schematic diagram of optimized scheduling for rolling forecasting of reservoirs.

From the perspective of operations research optimization, the real-time rolling forecast optimization scheduling model of the reservoir continuously executes the incoming water forecast, optimization solution, and scheduling decision-making process on a rolling basis, and each optimization solution model is equivalent to the deterministic optimization model. The process of real-time rolling forecast optimization of reservoir scheduling is illustrated in Fig. 4.The steps of real-time rolling forecast optimal scheduling for a reservoir with scheduling period $T$ and effective foresight period $t$ are as follows: ① make $t = 0$; ② the current time period is $t$, and obtain the forecasted water inflow in the future $t \to t + \tau$ time period; ③ according to the objective function and constraints, build a real-time rolling forecast optimal scheduling model for the reservoir in the future $t \to t + \tau$ time period, and then solve the model to obtain the optimal decision-making process in the future $t \to t + \tau$ time period; ④ According to the current optimal decision-making process in the future time period $t \to t + \tau$, assist in carrying out the reservoir scheduling operation facing the time period $t \to t + \tau$; ⑤ make $t = t + 1$; ⑥ if $t < T$, jump to step ②, otherwise, execute step ⑥; ⑦ reservoir scheduling to complete the task of the scheduling period, the end of the calculation, and the output of scheduling results. In summary, the specific implementation process is shown in Fig. 5.

### Establishment of rolling optimization scheduling model with forecasting model

In reservoir optimisation, runoff prediction data are core inputs, with prediction uncertainty and forecast horizon directly affecting the scheduling effectiveness. The hydrological forecasting system is updated periodically, generating future runoff forecasts based on current watershed storage, measured rainfall, and periodic predicted data. It is iteratively revised via real-time hydrological correction, and uncertainty increases with extended horizons, whereas periodic updates reduce bias.

The coupled rolling forecast optimisation scheduling model relies on dynamic time-step updates of hydrological data, with runoff prediction deviations decreasing as the information improves. During the scheduling period, the core strategy is to determine the current-stage plan using data within the forecast horizon, and revise subsequent plans with real-time updated forecasts (See Fig. 5). The specific process includes: constructing a mathematical model based on the current runoff prediction during the time period to generate a scheduling plan, and synchronously implementing the water release strategy for that time period; ① In the face of a time period $w$, a system optimization model is constructed based on available runoff prediction data to generate a discharge plan for that period and execute it. ② In the face of a time period $w + 1$, updated rainfall prediction and hydrological monitoring data are integrated to correct the runoff forecast results; The above two steps are continuously repeated until the scheduling period ends.

Rolling forecast optimization scheduling is based on the runoff information from time period 1 to $\tau$ (forecast period), and a comprehensive analysis of the scheduling benefits from time period 1 to $T$ (scheduling period) is conducted to determine the scheduling decision for time period 1 (decision period). The optimization scheduling model with the goal of maximizing flood control benefits during period 1 to $\tau$ can be expressed as Eq. (10), and the optimization scheduling model with the goal of maximizing power generation benefits can be expressed as Eq. (11). The decision variables are $[s_{1} ,s_{2} ,...,s_{H + 1} ]$, and the objective function is to maximize flood control and power generation benefits during period 1. The constraints include water balance conditions, water level and storage capacity constraints, discharge capacity constraints, and reservoir boundary constraints.

### Problem solving

The real-time rolling-forecast optimal scheduling of reservoirs is a typical nonlinear complex optimisation problem, for which this study selected the POA. Suitable for multistage dynamic decision-making with simple implementation steps, the POA adopts a priority method to handle the dual objectives of flood control and power generation. Additionally, the degree of constraint violation is treated as an objective, with multiple complex constraints addressed by minimising this degree of violation. In summary, the prioritization order is: first minimize constraint violation degree, then minimize maximum discharge flow, and finally maximize power generation during the scheduling period, and the implementation steps of the optimal scheduling model of real-time rolling forecast for the reservoir in time period $t_{0} \to t_{0} + \tau$ are as follows:

① know the outflow discharge $Q_{t0}^{{}}$ and the reservoir capacity $V_{t0}^{{}}$ in the initial moment of the facing time period $t_{0}$, and the number of scheduling time periods $\tau$;

② Let $n$ = 0, set the initial solution, take the flow as the decision variable as an example, the initial process optimal solution is recorded as ${\mathbf{Q}}_{{t_{0} \to t_{0} + \tau }}^{n} = \left\{ {Q_{{t_{0} }}^{n} ,Q_{{t_{0} + 1}}^{n} , \ldots ,Q_{{t_{0} + \tau }}^{n} } \right\}$, and calculate the corresponding constraint, flood control and power generation objective function values of $Q_{{t_{0} \to t_{0} + \tau }}^{n}$;

③ Set the global optimal solution of generation $n$, ${\mathbf{Q}}_{g}^{n} = {\mathbf{Q}}_{{t_{0} \to t_{0} + \tau }}^{n}$;

④ Make $$t = t_{0}$$;

⑤ Fix the outflow discharge of ${\mathbf{Q}}_{{t_{0} \to t_{0} + \tau }}^{0}$ at all times except the end of time period $t$, and the outflow discharge $Q_{t}^{n}$ at the end of temporary period $t$ on the opposite side is optimized in the decision space, and the optimization result is determined by the priority method (constraint objective > flood control objective > power generation objective), and the optimal $n + 1$ generation of $Q_{t}^{n + !}$ is obtained in the current situation;

⑥ Make $$t = t{ + }1$$;

⑦ if $t \le t_{0} + \tau$, skip step ⑤, otherwise skip step ⑧;

⑧ ${\mathbf{Q}}_{{t_{0} \to t_{0} + \tau }}^{n}$ through a round of traversal of the search for the optimal access to the ${\mathbf{Q}}_{{t_{0} \to t_{0} + \tau }}^{n + 1}$, the same using the priority method to compare the ${\mathbf{Q}}_{{t_{0} \to t_{0} + \tau }}^{n + 1}$ and ${\mathbf{Q}}_{g}^{n}$, if the ${\mathbf{Q}}_{{t_{0} \to t_{0} + \tau }}^{n + 1}$ is better than the ${\mathbf{Q}}_{g}^{n}$, so that the $n = n{ + }1$, skip step ③, otherwise skip step ⑨;

⑨ the end of the computation, the output of the optimal solution of ${\mathbf{Q}}_{g}^{n} = {\mathbf{Q}}_{{t_{0} \to t_{0} + \tau }}^{n}$.

**Fig. 6:**
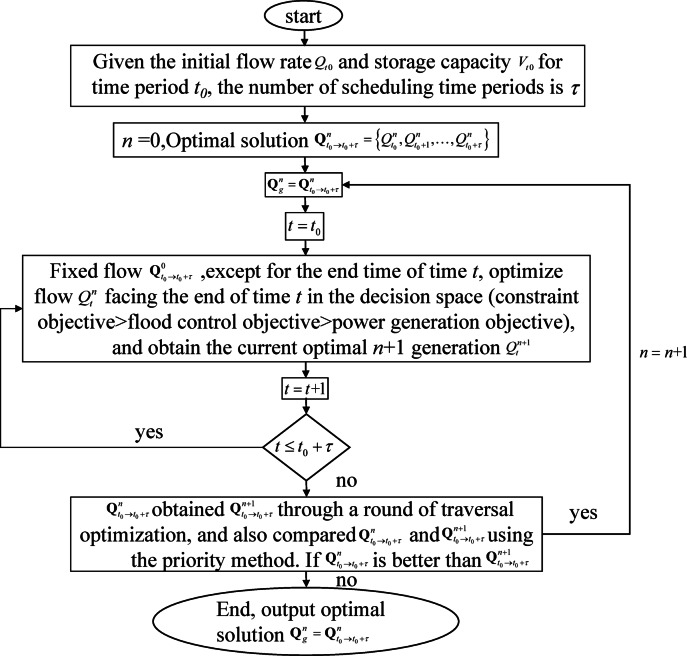
Implementation steps diagram of real-time rolling forecast optimization scheduling model for reservoirs.

The schematic diagram of the process steps is shown in Fig. 6.

## Case study

### Overview of the study area

**Fig. 7:**
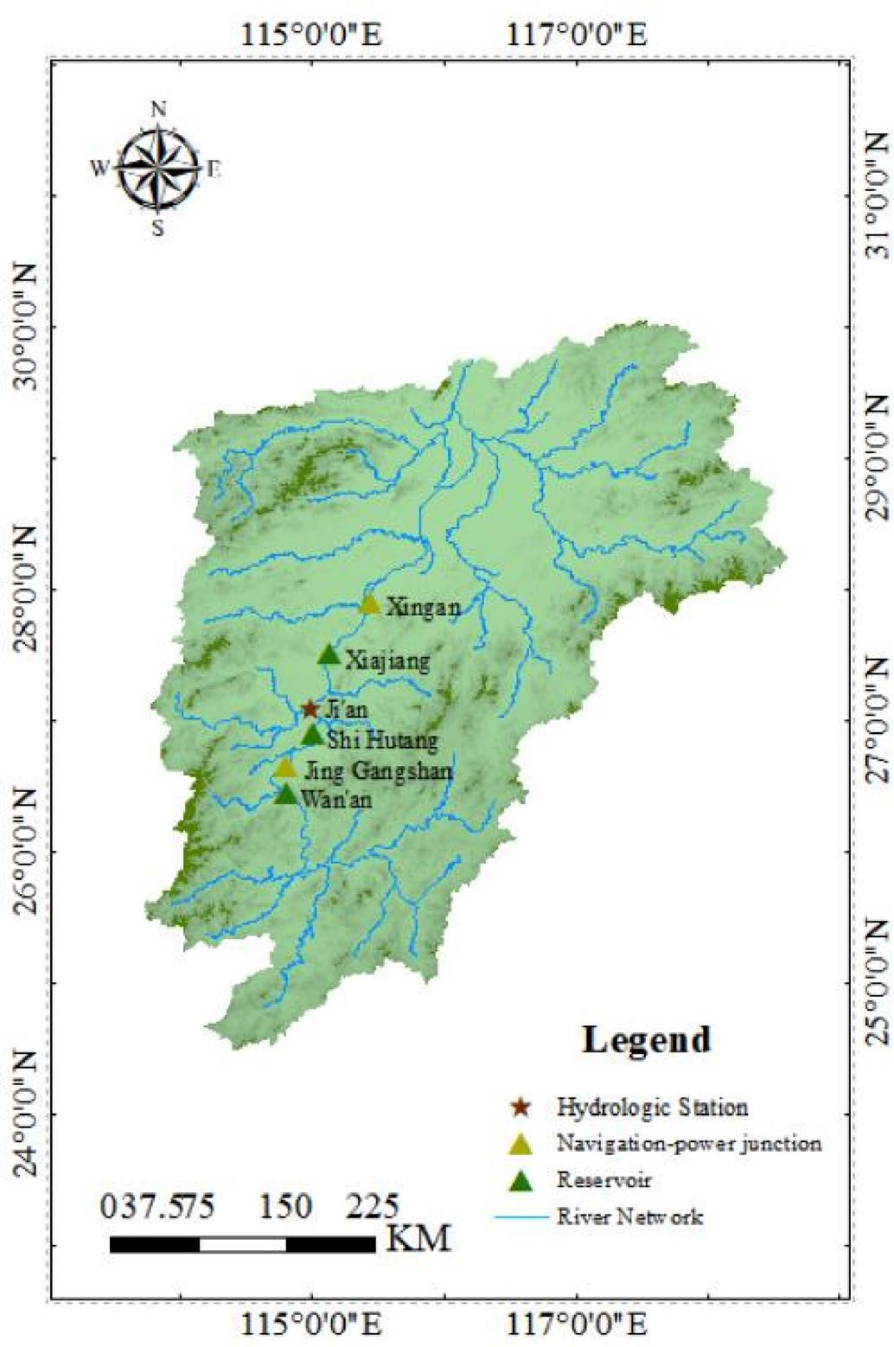
Xiajiang reservoir location map.

Xiajiang Reservoir is located in the central Ganjiang River, upstream of the former Xiajiang County seat. Serving multiple purposes including irrigation, power generation, transportation, and flood control, its key parameters are as follows: standard storage level 46.0 m, dead water level 44.0 m, flood control high water level, design flood level, and calibrated flood level all 49.0 m. The dam controls a 62,710 km^2^ basin, with an average outflow discharge of 1640 m^3^/s, runoff depth of 826.4 mm, and total runoff of 517.51 billion m^3^. The hydropower station has an installed capacity of 360 MW, annual power generation of 1.142 billion kWh, and irrigates 329.5 million m^2^. The reservoir has a total storage capacity of 11.87 billion m^3^, including 6.0 billion m^3^ of flood control storage and 2.14 billion m^3^ of exclusive flood control storage. The hub consists of spillway gates, a retaining dam, a riverbed power plant, a lock, left and right-bank irrigation inlet gates, and fish passes (see Fig. 7 for its geographical location).

### Calculation conditions for case study analysis

**Fig. 8:**
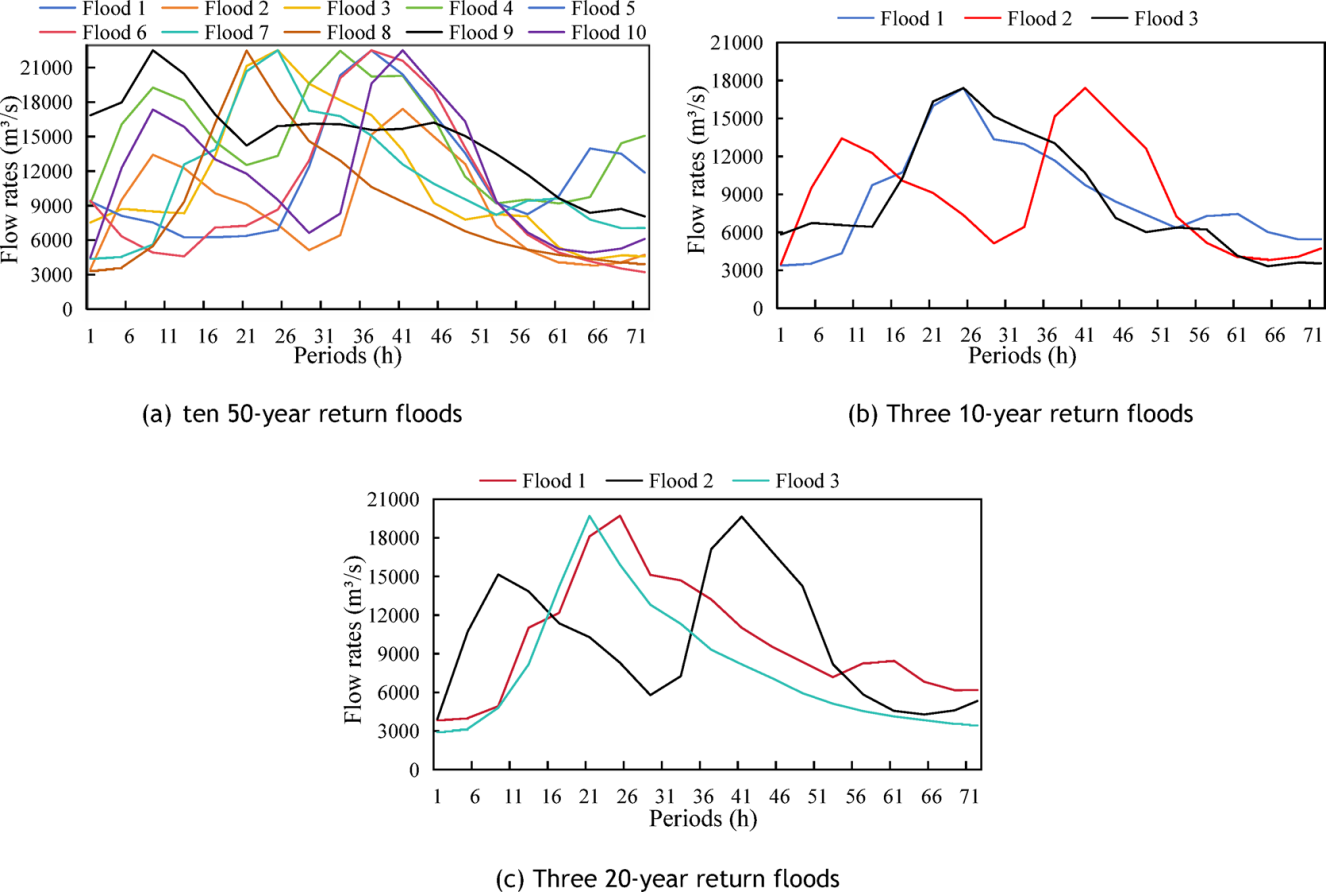
Flooding processes used for the calculation of 16 fields.

Xiajiang Reservoir’s downstream design flood control standard is 50-year return period. To account for different flood magnitudes, 16 typical design floods were chosen: 10 of 50-year, 3 of 20-year, and 3 of 10-year return periods were selected. Using the control variable method, this study focused on the flood forecast horizon and dynamic flood season water level control upper limit. The upper limits were 43.5, 44.0, 44.5, 45.0, 45.5, and 46.0 m; the forecast periods correspond to 12 h, 18 h, 24 h, 36 h, 48 h, and 72 h (6 h time step, 2, 3, 4, 6, 8, and 12 time periods, respectively). The design flood process lines and associated flood levels/peak flows are shown in Fig. 8 and Table 1.

**Table 1:** 16 field floods.

Number of floods	Magnitude	Maximum incoming flow (m^3^/s)
Flood 1	Floods of 50-year flood	22,500
Flood 2	Floods of 50-year flood	22,500
Flood 3	Floods of 50-year flood	22,500
Flood 4	Floods of 50-year flood	22,500
Flood 5	Floods of 50-year flood	22,500
Flood 6	Floods of 50-year flood	22,500
Flood 7	Floods of 50-year flood	22,500
Flood 8	Floods of 50-year flood	22,500
Flood 9	Floods of 50-year flood	22,500
Flood 10	Floods of 50-year flood	22,500
Flood 11	Floods of 10-year flood	17,500
Flood 12	Floods of 10-year flood	17,500
Flood 13	Floods of 10-year flood	17,500
Flood 14	Floods of 20-year flood	19,700
Flood 15	Floods of 20-year flood	19,700
Flood 16	Floods of 20-year flood	19,700

### Comparison and analysis of forecasting effects between different forecasting methods

This section presents a comparative analysis of the forecasting effects of six single forecasting models and four forecasting methods. Use the data from 0:00 on September 10, 2013 to 0:00 on May 22, 2022 as the training set, and the data from 6:00 on May 22, 2022 to 18:00 on July 24, 2024 (the last 20%) as the test set. After training and testing each model, the model for 12 step prediction with a prediction step size of 6 h. Unified error indicators, namely RMSE, MAE, DC, and QR, were used for comprehensive analysis and comparison.

### Single model performance comparison

To intuitively and systematically compare the performance of a single model, the six models (SVM, BP, LSTM, RT, GPR, and MLR) used in this study are summarised. A comparison of the prediction performances of the six models is presented in Table 2.

**Table 2:** Comparison of prediction effects of six models.

Model	RMSE	MAE	DC	QR
SVM	1010.69	495.36	0.67	0.45
BP	1030.81	498.92	0.66	0.47
LSTM	1057.89	687.32	0.64	0.26
RT	1103.74	570.22	0.60	0.42
GPR	767.04	337.54	0.81	0.64
MLR	988.69	509.82	0.69	0.43

After a comprehensive analysis of various models, distinct performance differences emerge. Here is a detailed breakdown of how each model fares in different aspects:

1. GPR performed optimally across all indicators: RMSE was 767.04, MAE was 337.54, DC was 0.81, and QR was 0.64. Its probabilistic framework and dynamic kernel function excel in noise suppression and non-linear modelling.
2. RT shows the weakest performance: RMSE reaches 1103.74 and MAE is 570.22, owing to the lack of a time-series memory mechanism.
3. BP achieves medium performance, with QR at 0.47, which is superior to SVM’s 0.45 and LSTM’s 0.26, demonstrating its hidden layer’s ability to capture nonlinear relationships.
4. MLR also performed moderately, with an RMSE of 988.69, but was limited by linear assumptions, resulting in a lower QR of 0.43.
5. Despite its theoretical advantages, LSTM shows no significant superiority: its comprehensive RMSE is 1057.89 and QR is 0.26, possibly due to data size and hyperparameter sensitivity. Overall, performance differences reflect the adaptation between model characteristics and data features: GPR’s probabilistic modeling suits complex hydrological time series, while static and linear models lack adaptability in dynamic prediction.

### Comparison of four forecasting methods

As mentioned earlier, the single model clock GPR model has the best prediction performance. To visually and clearly compare the performance of the single model, series coupling model, parallel coupled model, and series parallel coupled model, specific comparison results are shown in Table 3.

**Table 3:** Performance comparison of four prediction methods.

Model	RMSE	MAE	DC	QR
GPR	767.04	337.54	0.81	0.64
Parallel model	717.33	314.03	0.82	0.66
series coupling model	1534.07	675.03	0.42	0.43
series—parallel coupling model	774.65	373.77	0.80	0.58

A comparison of the error metrics among the single-model GPR, parallel model, series coupling model, and series–parallel model revealed notable differences.

1. The Single-model GPR performed stably, with an RMSE of 767.04, MAE of 337.54, DC of 0.81, and QR of 0.64.
2. The parallel model outperforms GPR across all indicators: RMSE is 717.33, MAE is 314.03, DC is 0.82, and QR is 0.66, which significantly boosts the prediction accuracy.
3. The series coupling model was the weakest, with an RMSE of 1534.07, MAE of 675.03, DC of 0.42, and QR of 0.43, owing to the limitations of data handling.
4. The series–parallel model was slightly inferior to the GPR and parallel models, with an RMSE of 774.65, MAE of 373.77, DC of 0.80, and QR of 0.58. It avoids the poor performance of the series model but fails to optimise further.

In summary, the parallel model is optimal, followed by the single-model GPR, while the series coupling model is worst and the series–parallel model offers limited advantages.

## Key factor analysis of reservoir scheduling with runoff forecasting

### Study on the influence mechanism of flood forecast horizon on reservoir scheduling efficiency

**Fig. 9:**
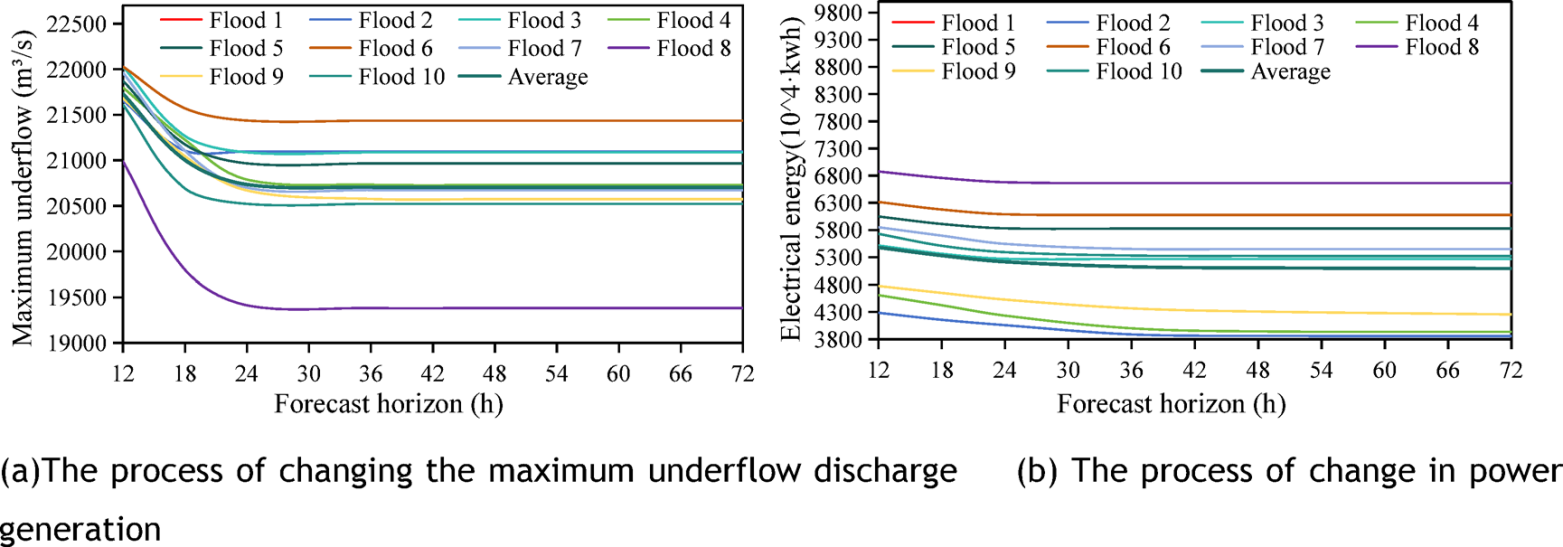
Variation of maximum underflow and power generation in different forecast horizon when the upper limit of dynamic flood control water level of 10 fields is 43.5 m.

This section designates the flood forecast horizon as the control variable for the study, with the flood limit water level remaining static and solely considering a flood limit water level of 43.5 m. Ten fields with a 50-year standard design flood were chosen as inputs, with a flood peak outflow discharge of 22,500 m^3^/s. 9 illustrates the variations in the maximum outflow discharge and power generation during the scheduling period as the flood forecast horizon $t$ increases. Analysis results show:

1. The maximum outflow discharge stabilises after a 24h forecast horizon. The average peak shaving efficiency is 3.42% at 12h, 6.66% at 18h, 7.85% at 24h, and remains steady at 7.98% thereafter.
2. Power generation decreased with extended forecast horizons, with peak reduction reaching 2,631,200 kWh and a 4.8% drop. It stabilizes after 3h, with only a 0.64% variation.
3. This trend stems from flood control prioritization: longer forecast horizons prompt advance reservoir emptying, lowering operating water levels, and reducing power generation head, as shown in Fig. 9.

### Analysis of the regulatory effect of dynamic flood control water level upper limit on reservoir scheduling benefits

**Fig. 10:**
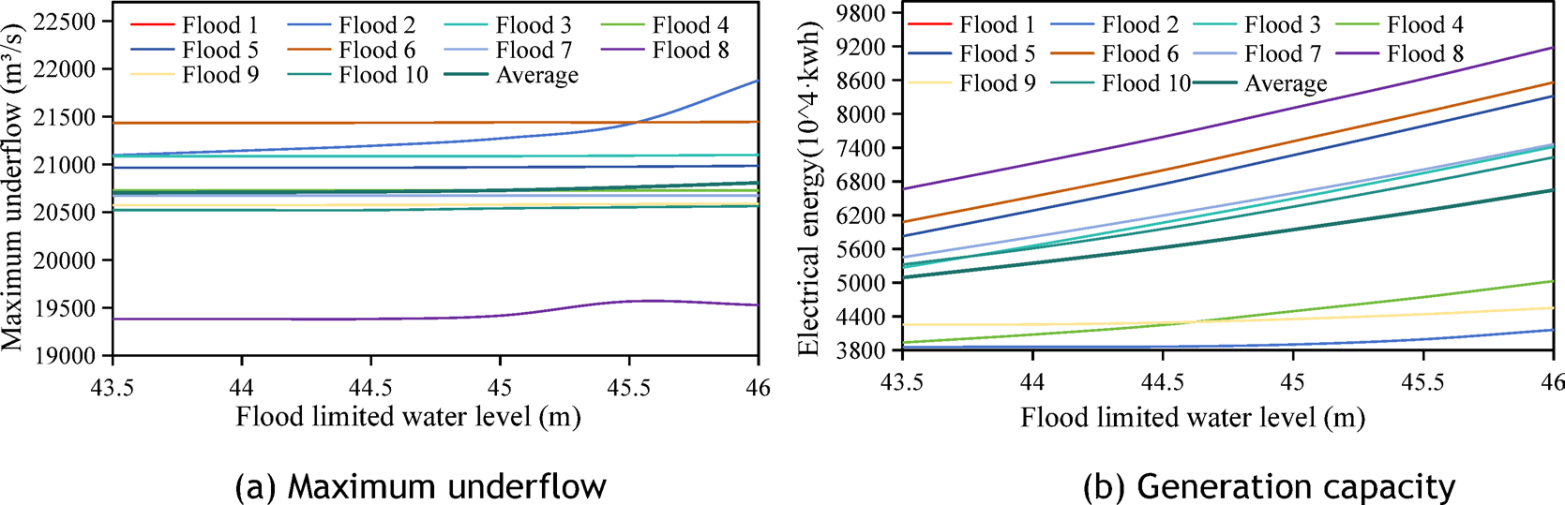
Changes in maximum discharged flow and power generation at different upper limit of dynamic flood control water level for the 10-field flood forecast horizon of 72 h.

This section analyzes the upper limit of the dynamic flood control water level during the flood season using 10 50-year return period standard-design floods with a 72-h forecast horizon. As shown in Fig. 10, the key findings are as follows:

1. The 72-h forecast enables rapid pre-discharge, with different upper limits of dynamic flood-control water levels, leading to lower flood-limited water levels, effectively controlling the impact of water level changes on the maximum discharge flow.
2. Power generation correlates significantly with the upper limit: each 0.5-m increase boosts power generation by an average of 3.11 million kWh, representing a 5.48% average increase. Comparing the 43.5 m and 46 m upper limits, the latter generates 15.56 million kWh more with a 30.55% increase, whereas the average peak-shaving rate is only 0.46% lower, showing obvious power generation gains.
3. A higher dynamic flood control water level increases the head difference before and after floods, enhancing power production despite the initial lower generation head, reflecting the flood control-power generation balance of the reservoir.

In summary, the 72-h forecast horizon effectively regulated the maximum discharge flow. Raising the upper limit of dynamic flood control water level significantly increases Xiajiang Reservoir’s power generation with minimal impact on flood control capacity. The corresponding relationship between water level and storage capacity is shown in Table 4.

**Table 4:** Table of water level reservoir capacity.

water level(m)	43.5	44.0	44.5	45	45.5	46
Storage capacity(billion m^3^)	4.43	4.88	5.36	5.88	6.43	7.02

### Combined analysis of the effects of forecast horizon and flood water level on optimal reservoir scheduling

**Fig. 11:**
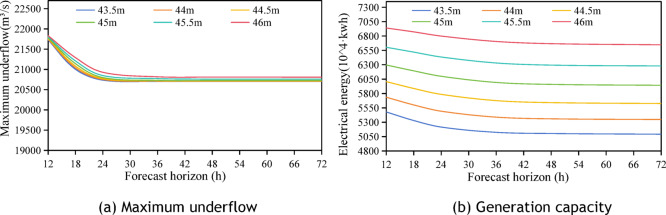
Effects of different the upper limit of dynamic flood control water level and different forecast horizon on maximum discharge flow and power generation.

This section analyzes the impact of the upper limit of dynamic water management levels during the flood season, in conjunction with the flood forecast horizon. The average maximum discharge flow and average power generation under various control factors are illustrated in Fig. 11, based on the standard design flood with a 50-year return period. The analysis results indicate the following.

1. The maximum outflow discharge diminishes as the flood forecast period extends, remaining relatively constant after 24 h.
2. The maximum outflow discharge increases with an increase in the upper limit of dynamic flood water level control.
3. Power generation declines with a longer forecast period and stabilizes after 36 h.
4. Power generation increases with an increase in the upper limit of dynamic flood control level.

### Exploration of the correlation effect of flood magnitude differences on reservoir scheduling effects

**Fig. 12:**
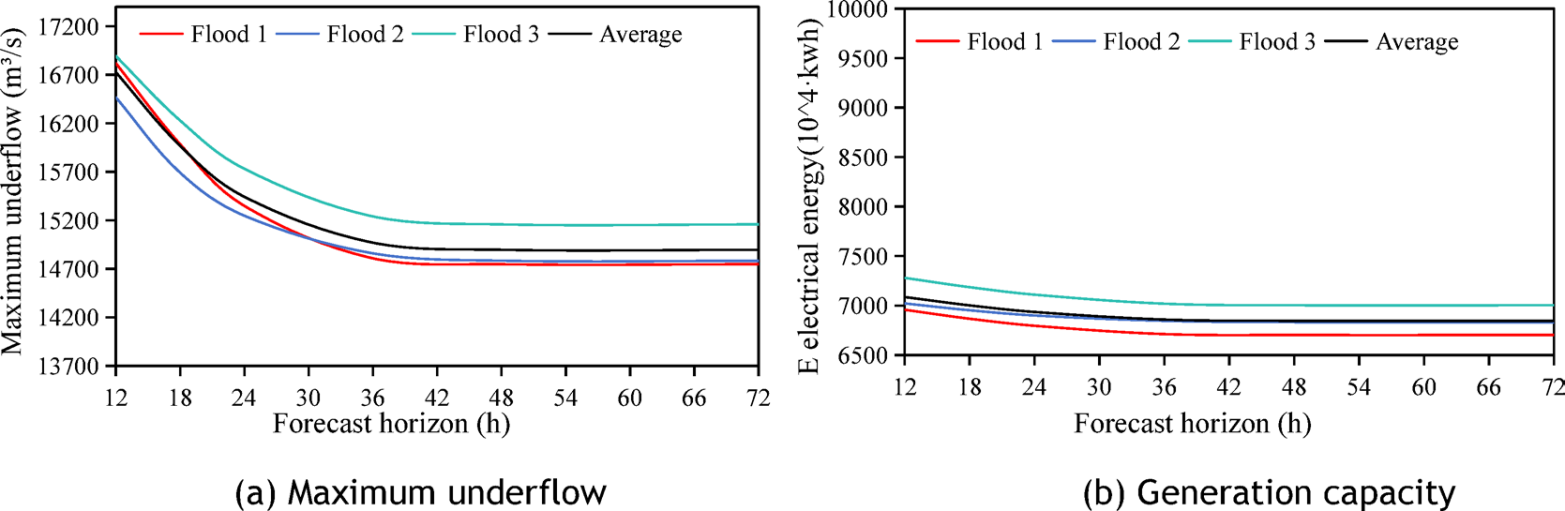
Effect of different forecast horizons on the maximum discharge flow and power generation when the upper limit of dynamic flood control water level is 43.5 m for the 10-year return flood.

**Fig. 13:**
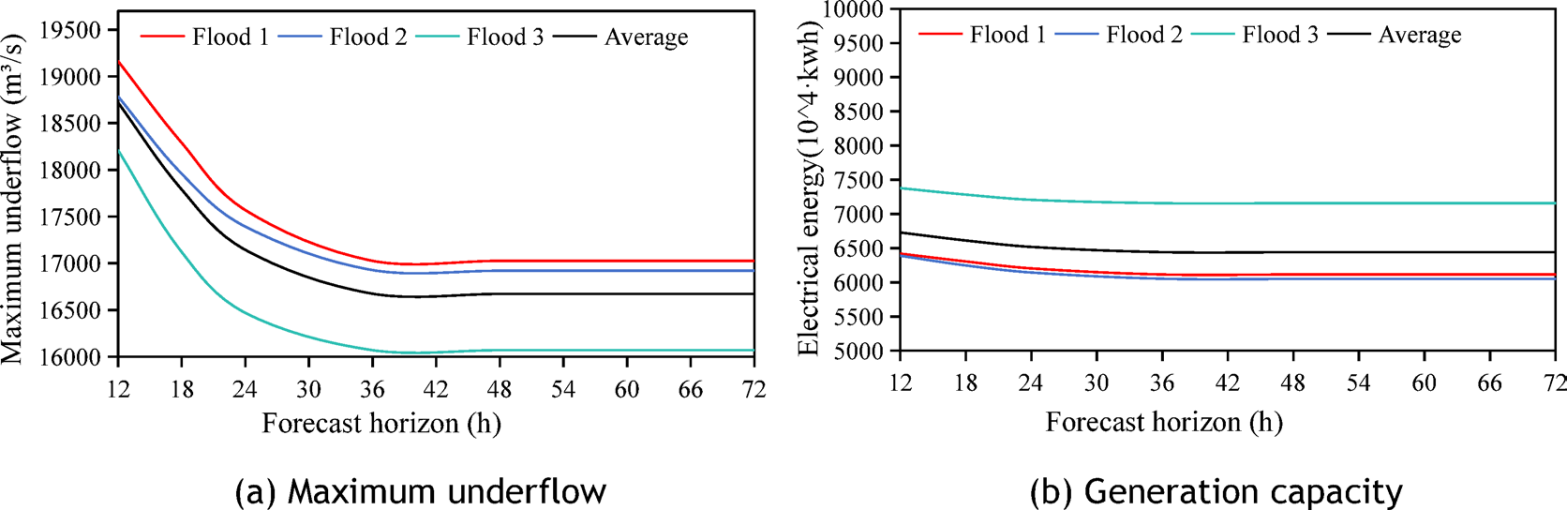
Effect of different forecast horizons on the maximum discharge flow and power generation when the upper limit of dynamic flood control water level is 43.5 m for a 20-year return flood.

This section analyzes the flood forecast horizon with a flood threshold water level fixed at 43.5 m. It focuses on 10-year and 20-year return period standard design floods, with peak flows of 17,400 m^3^/s and 19,700 m^3^/s, respectively, and combines 50-year return period data. The evolution of the average maximum discharge flow and average power generation with extended forecast horizons is shown in Figs. 12 and 13, respectively.

1. Flood magnitude is inversely related to the optimal peak-shaving forecast horizon; higher flood magnitudes require shorter horizons. The optimal horizons for 50-year, 20-year, and 10-year return floods were 24h, 36h, and 42h respectively, with corresponding maximum peak shaving rates of 7.85%, 15.37%, and 14.39%.
2. The Xiajiang Reservoir has limited flood control capacity and storage space; therefore, shorter forecast horizons are required for large floods to retain floodwaters. For small floods, sufficient storage allows longer horizons for preemptive discharge, reflecting the interaction between reservoir storage conditions and the flood magnitude.

In summary, the flood forecast horizon is closely related to flood magnitude and reservoir characteristics. Clarifying this relationship is crucial for optimizing flood control and peak-shaving, enhancing reservoir operation efficiency and safety.

**Fig. 14:**
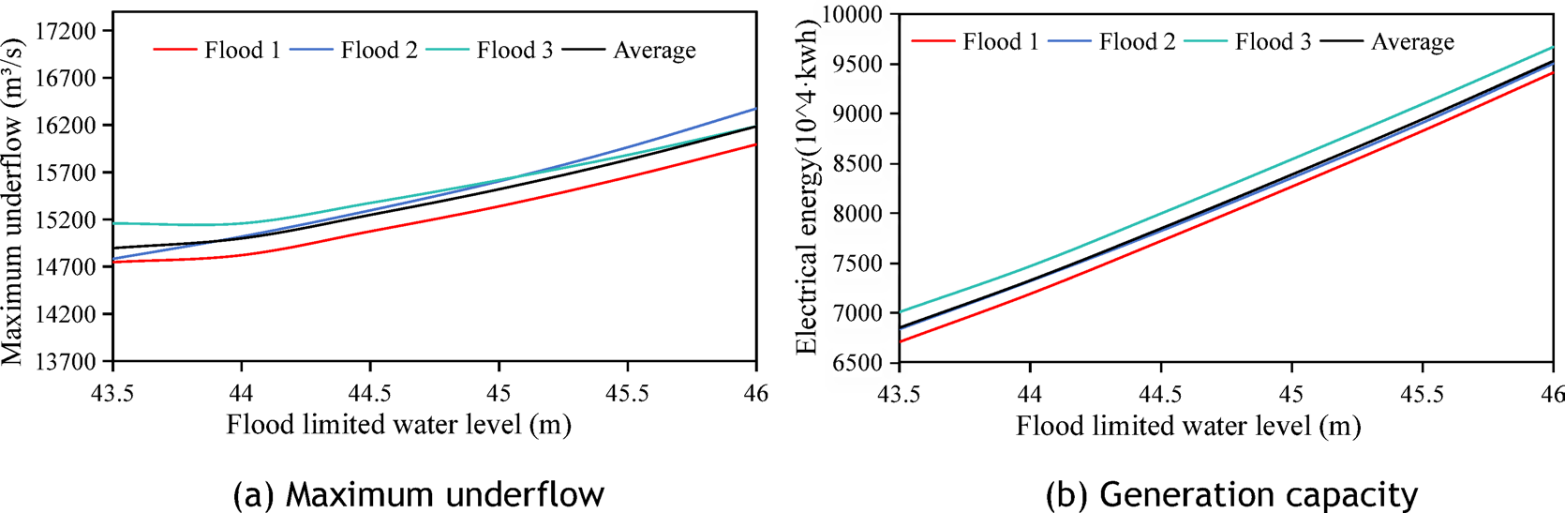
Changes in maximum discharge flow and power generation at different upper dynamic flood control water level for a 72 h forecast horizon of 10-year return flood at 3 sites.

**Fig. 15:**
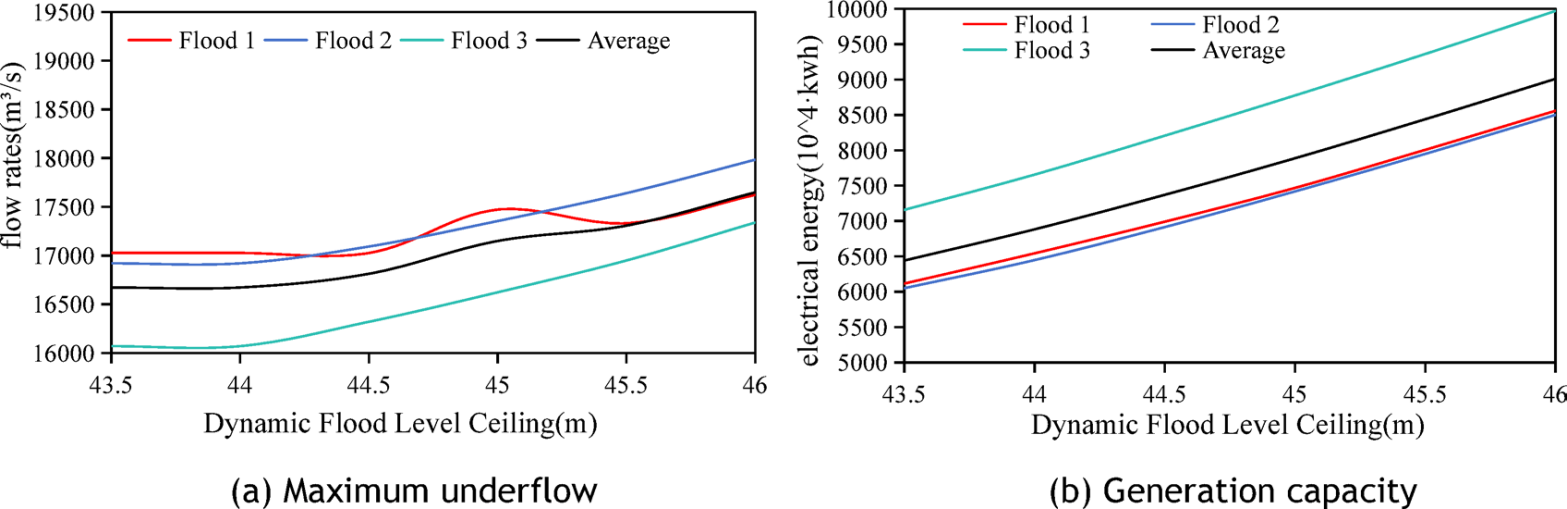
Changes in maximum discharged flow and power generation at different upper dynamic flood control water level during the 72 h forecast horizon of 20-year return flood for the 3- field.

Taking the maximum dynamic flood control water level as the control variable, the analysis focuses on the 72 h forecast horizon, with the experimental results of the 10-year and 20-year return period standard-design floods shown in Figs. 14 and 15, respectively. Key findings: Flood magnitude was inversely correlated with dispatch-period power generation. With a 43.5 m flood limit water level and 72 h forecast, the average power generation is 50.91 million kWh (50-year flood), 64.42 million kWh (20-year), and 68.46 million kWh (10-year). Higher flood magnitude increases inflow and discharge flow, raising tailwater level, reducing power generation head and thus hydropower output.

### Analysis of typical historical inflow process

To further verify the applicability and reliability of the multi-model series–parallel coupling correction framework in actual hydrological scenarios, this study selected four typical real inflow processes for empirical analysis. These processes cover two key scenarios: high-flow and low-flow. High-flow processes 1 and 2 correspond to large flood events, whereas low-flow processes 1 and 2 represent inflow states during normal and dry periods, comprehensively testing the prediction accuracy of the model under different runoff conditions. Based on the established prediction model system, four methods—GPR single model, parallel coupling model, series coupling model, and series–parallel coupling model—were used for runoff prediction, with a comprehensive evaluation conducted using four core error indicators: RMSE, MAE, DC, and QR. The error indicators of the four forecasting methods for the two floods are listed in Table 5.

**Table 5:** Comprehensive effect comparison of forecast process evaluation results for four forecasting techniques in flood cases 1 and 2.

	Forecasting technology	RMSE	MAE	DC	QR
Flood 1	GPR single model	2571.05	1820.83	0.26	0.52
	Parallel coupling model	2352.92	1652.40	0.42	0.54
	Series coupling model	5142.11	3641.67	− 1.98	0.42
	Series parallel coupling model	4705.83	3304.80	− 1.31	0.36
Flood 2	GPR single model	1894.33	1111.12	0.46	0.64
	Parallel coupling model	1605.82	971.08	0.65	0.67
	Series coupling model	3788.65	2222.24	− 1.16	0.54
	Series parallel coupling model	3211.64	1942.17	− 0.38	0.46

The results in Table 5 show that the GPR single model and parallel coupling model perform strongly in both Flood 1 and Flood 2, whereas the series coupling model and series–parallel coupling model perform poorly.

In Flood 1, the GPR single and parallel coupling models had relatively low RMSE and MAE values. The GPR single model’s RMSE was 2571.05, and MAE was 1820.83, with DC 0.26 and QR 0.52. Parallel coupling model’s RMSE is 2352.92 and MAE is 1652.40, with DC 0.42 and QR 0.54, indicating acceptable consistency and qualification with actual inflow.In Flood 2, both models maintain good performance: GPR single model has RMSE 1894.33, MAE 1111.12, DC 0.46 and QR 0.64; parallel coupling model has RMSE 1605.82, MAE 971.08, DC 0.65 and QR 0.67.In contrast, the series coupling model performed the worst. Its RMSE and MAE were more than double those of the GPR and parallel models, and the DC was negative — -1.98 in Flood 1 and -1.16 in Flood 2). Series–parallel coupling model also lags significantly, with much higher RMSE and MAE, and lower DC and QR than GPR and parallel models.

The analysis in Table 6 for Low Inflow Cases 1 and 2 shows that the GPR single model and parallel coupling model maintain a strong forecasting performance, while the series coupling model remains poor. The performance gap narrows compared to that in flood cases, and the series–parallel coupling model exhibits better adaptability.

**Table 6:** Comprehensive effect comparison of forecast process evaluation results for four forecasting techniques in low inflow cases 1 and 2.

	Forecasting technology	RMSE	MAE	DC	QR
Low Inflow 1	GPR single model	494.93	316.69	0.72	0.68
	Parallel coupling model	480.77	316.79	0.75	0.72
	Series coupling model	745.79	446.16	0.30	0.68
	Series parallel coupling model	520.81	263.27	0.70	0.80
Low Inflow 2	GPR single model	404.98	238.01	0.69	0.80
	Parallel coupling model	395.68	248.56	0.75	0.81
	Series coupling model	643.40	441.61	0.39	0.67
	Series parallel coupling model	542.12	312.05	0.61	0.71

In Low Inflow 1, the GPR single model had an RMSE of 494.93, MAE of 316.69, DC of 0.72, and QR of 0.68; the parallel coupling model had an RMSE of 480.77, MAE of 316.79, DC of 0.75, QR 0.72. The series coupling model lags with RMSE 745.79, MAE 446.16, and DC 0.30; the series–parallel model performs well with QR 0.80 and DC 0.70.

In Low Inflow 2, the GPR single model has an RMSE of 404.98, MAE of 238.01, DC of 0.69, and QR of 0.80; the parallel coupling model has an RMSE of 395.68, MAE of 248.56, DC of 0.75, QR 0.81. Series coupling model underperforms with RMSE 643.40, MAE 441.61, DC 0.39; series–parallel model narrows the gap with DC 0.61, QR 0.71.

### Analysis of forecast scheduling results of real water process and comparison with historical dispatching process

To assess the model’s practical flood scheduling effectiveness, four inflow processes of varying magnitudes were tested, and the outputs were compared with the actual records of the Xiajiang Reservoir. Owing to the poor performance of the series and series–parallel models, only the GPR single model and parallel-coupled model were analysed.

Table 7 presents the scheduling effects of the two high-flow processes under the four methods, with a 72-h forecast horizon and 43.5 m dynamic water level upper limit. All methods performed unsatisfactorily versus historical records: High Flow Process 1 had a historical maximum discharge of 11,327.62 m^3^/s and a 13.08% peak shaving rate, and High Flow Process 2 had 9,851.29 m^3^/s and 14.05%. All forecast methods showed higher discharge and lower peak shaving rates, the lowest being -5.37%. The parallel coupled model slightly outperforms GPR: in Process 1, it has 84.01 m^3^/s lower discharge and 0.65 pp higher peak shaving rate; in Process 2, it has 771.78 m^3^/s lower discharge and 6.73 pp higher peak shaving rate.

**Table 7:** Comparison of scheduling results of four prediction methods for high flow processes 1 and 2.

		Maximum discharge flow(m^3^/s)	Power generation (10,000 kWh)	Peak shaving rate (%)
High flow process 1	Historical scheduling results	11,327.62	7,541.87	13.08
	GPR single forecast model	12,890.86	10,293.39	1.08
	Coupled parallel forecast model	12,806.85	11,099.5	1.73
	GPR series forecast model	13,732.26	9,666.11	− 5.37
	Coupled series–parallel forecast model	14,657.26	11,100.88	− 4.80
High flow process 2	Historical scheduling results	9,851.29	8,077.87	14.05
	GPR single forecast model	11,122.71	9,911.26	2.96
	Coupled parallel forecast model	10,350.93	10,289.21	9.69
	GPR series forecast model	11,843.68	9,603.84	− 3.33
	Coupled series–parallel forecast model	10,422.5	10,332.09	0.35

Table 8 compares the scheduling results of the four prediction methods for the two low-flow processes. The GPR single-forecast and coupled parallel forecast models achieved good results, with significant power generation increases compared to the historical results. For Low inflow Process 1, historical power generation is 1,582.27 thousand kWh, with the two models yielding 1,661.36 and 1,683.28 thousand kWh respectively; for Process 2, historical output is 6,079.44 thousand kWh, and the two models produce 6,658.13 and 6,702.58 thousand kWh. The coupled parallel model performs slightly better, indicating the study’s forecast scheduling models are well-suited for low-flow scenarios.

**Table 8:** Comparison of scheduling results of four prediction methods for Low inflow processes 1 and 2.

		Power generation (10,000 kWh)
Low inflow process 1	Historical scheduling results	1,582.27
	GPR single forecast model	1,661.36
	Coupled parallel forecast model	1,683.28
	GPR series forecast model	1,602.77
	Coupled series–parallel forecast model	1,620.71
Low inflow process 2	Historical scheduling results	6,079.44
	GPR single forecast model	6,658.13
	Coupled parallel forecast model	6,702.58
	GPR series forecast model	6,615.86
	Coupled series–parallel forecast model	6,621.81

Observations on scheduling results under different prediction periods show longer horizons do not necessarily yield better effects, possibly due to poor inflow forecasting performance. Researchers thus compared scheduling results of four inflow processes with different flow magnitudes under two prediction periods: 24 h and 72 h. Table 9 presents results for high-flow processes 1 and 2, and Table 10 shows those for low-flow processes.

**Table 9:** Comparison of scheduling results of high flow processes 1 and 2.

	Forecasting method	Forecast horizon	Maximum discharge flow(m^3^/s)	Power generation (10,000 kWh)	Peak shaving rate (%)
High flow process 1	GPR single forecast model	24h	13,032.15	11,071.53	0.00
		72h	12,890.86	10,293.39	1.08
	Coupled parallel forecast model	24h	12,894.1	11,144.8	1.06
		72h	12,806.85	11,099.5	1.73
High flow process 2	GPR single forecast model	24h	12,648.72	9,860.37	1.89
		72h	11,122.71	9,711.26	2.96
	Coupled parallel forecast model	24h	10,350.93	10,311.27	9.69
		72h	10,350.93	10,289.21	9.69

**Table 10:** Comparison of scheduling results of Low inflow processes 1 and 2.

	Forecasting method	Forecast horizon	Power generation (10,000 kWh)
Low inflow process 1	GPR single forecast model	24h	1,785.29
		72h	1,661.36
	Coupled parallel forecast model	24h	1,797.04
		72h	1,683.28
Low inflow process 2	GPR single forecast model	24h	6,710.9
		72h	6,658.13
	Coupled parallel forecast model	24h	6,741.87
		72h	6,702.58

Table 9 presents the scheduling results of High Flow Processes 1 and 2 for the two forecasting methods and horizons (24 h, 72 h). Flood scheduling focuses on the peak shaving rate and maximum discharge flow, with longer horizons yielding better effects.For Process 1, both methods perform better at 72 h: the GPR single model’s maximum discharge drops from 13,032.15 to 12,890.86 m^3^/s, and the peak shaving rate rises from 0.00 to 1.08%; the coupled parallel model’s discharge falls from 12,894.1 to 12,806.85 m^3^/s, and the rate increases from 1.06 to 1.73%.For Process 2, the GPR single model’s discharge plummets from 12,648.72 to 11,122.71 m^3^/s, with a rate from 1.89 to 2.96%; the coupled parallel model maintains stability with 10,350.93 m^3^/s discharge and 9.69% rate across both horizons.Results confirm longer horizons improve high-flow scheduling against core flood control indicators.

Table 10 presents the scheduling results of the two low-flow processes for the two forecasting methods and horizons. Low-flow scheduling focuses on the peak shaving rate, maximum discharge flow, and power generation, reflecting the rationality of water utilisation, with longer horizons yielding worse effects.

For both processes, power generation declines at 72 h versus 24 h: Low inflow Process 1 sees GPR single model drop from 1,785.29 to 1,661.36 ten thousand kWh, coupled parallel model from 1,797.04 to 1,683.28 ten thousand kWh; Low inflow Process 2 has GPR single model fall from 6,710.9 to 6,658.13 ten thousand kWh, coupled parallel model from 6,741.87 to 6,702.58 ten thousand kWh.

This consistent reduction shows longer forecast horizons fail to optimise coordination between peak shaving, discharge control and water utilisation, worsening low-flow scheduling effects.

## Discussion

### Optimization strategy for flood control and power generation scheduling of Xiajiang Reservoir

This study focuses on the Xiajiang Reservoir and achieves key results in reservoir scheduling.

1. A multi-model series–parallel coupling inflow prediction model was developed, with the parallel framework and single GPR model outperforming series and series–parallel coupling models.
2. An integrated rolling prediction optimization scheduling model was constructed, leveraging dynamic hydrological data and POA algorithm to balance flood control and power generation.
3. Simulations of 16 typical design floods show maximum outflow stabilizes after 24h of forecast horizon, while moderately raising dynamic flood control water level boosts power generation (e.g., 30.55% increase from 43.5m to 46.0m) with minimal flood risk.
4. The integrated prediction-scheduling framework exhibits strong engineering applicability, providing a replicable technical path for similar reservoirs in the Yangtze River Basin.

Optimizing rolling-forecast scheduling requires balancing dual benefits, determining optimal forecast horizons and water levels, and enhancing prediction accuracy—laying a theoretical and practical foundation for reservoir operation.

### Limitations of the research in this paper

Although this study achieved significant results, some limitations affect the accuracy and reliability of the findings.

1. The research only involves design floods with 10-year, 20-year, and 50-year return periods, excluding extreme and small-to-medium flood scales. Their distinct characteristics may lead to different scheduling responses, limiting the model’s universal applicability. Future work should expand the flood scale coverage.
2. The study focuses primarily on forecast horizon and dynamic flood control water level, neglecting factors like climate change and land use change that influence reservoir inflow and outflow. This makes the model less reflective of the actual conditions, requiring integration of such factors in future optimisations.

Despite these limitations, this study significantly advances reservoir scheduling methodologies. Examining these constraints and proposing solutions will help improve the accuracy and reliability of future research.

### Future outlook and research recommendations

Future investigations can optimise the model’s accuracy and reliability through the following improvements.

1. Integrate physics-based hydrological models with AI to combine mechanism interpretability and data-fitting capability, compensating for single-model limitations and enhancing prediction robustness.
2. Extend the model’s applicability to other reservoirs by coupling with climate models, incorporating climate change impacts for better adaptation to long-term hydrological variations.
3. Explore geographical and climatic impacts on reservoir operations, explicitly considering inflow, prediction, and parameter uncertainties to develop adaptive flood control-power generation scheduling strategies.
4. Narrow the low- and high-flow performance gap by enriching high-flow samples, developing hybrid physics-data-driven models, and designing scenario-specific strategies.

These improvements may bring challenges such as mechanism-AI fusion, multi-reservoir coordination, climate model integration, multi-source uncertainty quantification and adaptive rule design, which require further investigation for future study expansion.

## Conclusion

This study took the Xiajiang Reservoir as the research object, constructed two key models, and analysed the impacts of key factors on flood control and power generation benefits using typical design floods. The main conclusions are as follows:

1. A multi-model series–parallel coupling inflow prediction model was developed. The parallel framework achieved excellent short-term runoff prediction with an RMSE of 717.33, DC of 0.82, and QR of 0.66, outperforming the optimal single GPR model, while the series coupling model performed poorly with an RMSE of 1534.07 and DC of 0.42.
2. A reservoir rolling prediction optimisation scheduling model was established, integrating dynamically updated hydrological data and the inflow prediction model via a rolling time window, with the POA algorithm for iterative optimisation to balance dual objectives in line with actual operation.
3. Using 16 typical design floods, the regulatory effects of the flood forecast period and dynamic flood control water level were clarified. Maximum outflow stabilised after 24 h with 7.98% peak-shaving efficiency within 72 h, and raising the water level upper limit from 43.5 to 46 m increased power generation by 30.55% with 0.46% lower peak-shaving rate.
4. Traditional historical data-dependent forecasting methods have limitations. Scarce high-flow samples lead to poor scheduling with peak shaving rates of 1.08 to 9.69%, while abundant low-flow samples ensure high accuracy, with the parallel model achieving an RMSE of 395.68 and 5.0 to 10.0% higher power generation than historical results.

## Supplementary Information

Supplementary Information.

## Data Availability

All data and code are available from the corresponding author.
